# American Dental Association

**Published:** 1888-01

**Authors:** 


### AMERICAN DENTAL ASSOCIATION.

### TWENTY-SEVENTH ANNUAL MEETING.

Reported for the Independent Practitioner.

Concluded from Page 641, Vol. VIII.

### THURSDAY EVENING SESSION.

A resolution was adopted setting aside one thousand dollars as a special fund, to be under the control of the Executive Committee and held for the protection of dentists against the unlawful demands of the possessors of dental patents.

Section VII was called—Anatomy, Pathology and Surgery—and the report was read by the Chairman, Dr. T. W. Brophy. A paper by Dr. Atkinson upon "Sponge Grafting" was offered, and the subject of Implantation recommended for discussion.

Dr. Atkinson's paper was read by Dr. Rhein. The sponge-graft, it asserted, is primarily adapted to the reproduction of tissue lost traumatically, or when there is no inflammation. The author had never seen a failure to unite by first intention. The final disposition of the sponge is still an open question.

His first case was three years ago, the external plate of the alveolus being lost, an extensive fistula existing. This was enlarged by tents, the carious portion of the bone burred out and the cavity washed with aromatic sulphuric acid and with styptic colloid, and treated to a spray of mercuric chloride. Then for three days the cavity was dressed with a sterilized dressing. The fourth day a piece of sterilized sponge a little larger than the chasm was prepared by placing it in a glass and pouring a solution of warm mercuric chloride upon it. It was wrung out and carefully inserted. The next day there was a slight effusion of serum which was removed by bibulous paper. The next day there were indications of blood in the graft. Two days subsequently the trabeculæ were filling up. At the next visit there were indications of pus where the sponge was not adherent. The parts which had not adhered were clipped out until the vascular part was reached, when it was sprayed with mercuric chloride. After a week there was another indication of pus manifest.

This was treated as before, when a scab formed, and it is now indistinguishable from the rest. This was a fair average case. The sterilization of the sponge is accomplished by heating it in mercuric chloride to 120° F. At least three times per day the case should be sprayed with the mercuric chloride.

*Dr. Rhein*—Said that he had been employing the sponge-graft with unvarying success. It will reproduce every kind of tissue, but the territory into which it is introduced must be in healthy condition. This is essential to success.

*Dr. Morrison*—Eleven years ago I presented a paper upon the replanting of teeth. One of the cases cited was exhibited to Dr. Younger last fall. Odontoclasts had once been at work, but preventive measures had kept the tooth until the present time. Daily friction on the root had cured the angry symptoms. He had not as much faith in the implantation of old teeth as of those freshly extracted, but Dr. Younger has, and this encouraged him to make trials.

*Dr. Harroun*—Had replanted a tooth that had been out for eight weeks. He was obliged to open up the socket, which was easily done with a twist drill, and the tooth was now giving good satisfaction.

Section I—Prosthetic Dentistry, Metallurgy and Chemistry—was called. The Chairman, Dr. Harroun, said there was no report, as the Secretary was not present and had sent nothing. He presented, however, a few subjects for discussion.

The subject of chemistry is too much neglected. We have few chemists, and what we have are not very active. Metallurgy has retrograded since the advent of rubber. We are below the standard of twenty-five years ago. Few of our leading men do anything in the laboratory, sending their work out to men who have no knowledge of the peculiar demands of the case. Crown and bridge-work is making advances, and there is no doubt it will fulfill a useful purpose in the hands of competent men. Aluminum gives promise of great usefulness. Dr. Carroll, of Meadville, Pa., has apparently overcome many of the obstacles to its general use. Metallic linings to vegetable plates present advantages in point of cleanliness, but they cannot take the place of metal plates.

*Dr. Hunt*—Said that heretofore aluminum had been impracticable in dentistry. It could not be satisfactorily soldered, nor had any method of casting it been a practical success. But to-day the

Association had seen true scientific work done in a clinic. It has been proved that all that is necessary is to keep the surfaces entirely clean when, under the influence of heat, they will unite. Its manipulation seems almost as easy as that of rubber. A little more experience and we shall be possessed of a metal that can be readily worked by any dentist. The demonstration of Dr. Carroll had delighted him, and he believed that it was the greatest advance in mechanical dentistry that this generation of dentists had seen.

It was moved and carried that the appropriation for sectional work be the same as last year.

It was moved and carried that Section II be directed to report at the next meeting a plan for a course of elementary instruction in Dental Histology, Anatomy and Hygiene, in the public schools.

The officers for the ensuing year were then installed. A vote of thanks to the retiring President for the dignity and ability which he had exhibited in the fulfillment of his duties was passed, and to Dr. S. A. Freeman for his untiring labors as chairman of the local Committee of Arrangements.

President Abbott appointed Drs. E. T. Darby and A. W. Harlan members of the Publication Committee, and Drs. W. W. Walker, M. W. Foster and T. T. Moore, members of the local Committee of Arrangements.

The Association then adjourned to meet in Louisville, Ky., in joint session with the Southern Dental Association, on the first Tuesday of August, 1888.

